# Linoleic acid addition prevents *Staphylococcus aureus* biofilm formation on PMMA bone cement

**DOI:** 10.1016/j.bioflm.2025.100311

**Published:** 2025-08-07

**Authors:** Linglu Hong, Karin Hjort, Dan I. Andersson, Cecilia Persson

**Affiliations:** a Division of Biomedical Engineering, Department of Materials Science and Engineering, Uppsala University, Uppsala, Sweden; b Department of Medical Biochemistry and Microbiology, Uppsala University, Uppsala, Sweden

**Keywords:** PMMA bone cement, linoleic acid, Antibiofilm, Methyl methacrylate monomer, Antibiotics

## Abstract

Acrylic bone cement is widely used in vertebroplasty to treat osteoporosis-induced vertebral compression fractures. However, infection after vertebroplasty is problematic and previous work has suggested loading the bone cement with an antibiotic for prophylaxis. Linoleic acid (LA) has been investigated as a promising additive to improve the mechanical properties of bone cement for vertebroplasty, but LA could potentially also have an antibacterial effect. In this study, we evaluated the antibacterial properties of LA-loaded bone cement by comparing its antibiofilm properties with that of original bone cement through quantification of bacterial growth using viable cell count and scanning electron microscopy. The released monomer (MMA) concentration and the monomer minimum inhibitory concentration were determined to clarify the monomer's potential role in inhibiting bacterial growth. The LA release profile was measured, and a checkerboard assay was done to determine any synergistic effects of LA and the commonly used antibiotic gentamicin. Results show that LA-loaded bone cement could significantly inhibit *Staphylococcus aureus* biofilm formation, including gentamicin-resistant strains, but with limited effect on *Escherichia coli*. Furthermore, the released MMA did not have a significant influence on bacterial growth. The checkerboard assay results show that the LA and gentamicin combination could broaden the antibacterial spectrum and increase gentamicin efficacy. In conclusion, LA merits further investigation as an antibacterial agent in bone cement, alone or in combination with antibiotics.

## 1 Introduction

Polymer-based polymethyl methacrylate (PMMA) bone cement is a moldable and injectable orthopedic biomaterial [1]. In the 1950s, the use of PMMA bone cement in the fixation of total hip replacements, at the time a new surgical technique, was introduced. In the 1970s, to prevent post-operation infections, the addition of antibiotics to bone cement was introduced [[1], [2], [3]]. Nowadays, PMMA bone cement is also used in vertebral augmentation procedures including vertebroplasty (VP), which is a minimally invasive surgical procedure for the treatment of osteoporosis-induced vertebral compression fractures [4]. In this procedure, VP stabilizes the fractured vertebral body and reduces pain [5].

Infection after VP, a rare but serious complication is a concern [6]. Liao et al. reported that 0.32 % of patients suffered infectious spondylitis after VP, and among them around 61 % resulted in mortality and/or disability after revision surgery [6]. Also, revision surgery to remove the cement from the vertebral body is a challenging task [7,8]. In particular, patients having a history of infection prior to VP carry a higher risk of suffering from infectious complications [6,9]. Another important issue with infections is the potential formation of biofilm that complicates treatment [10]. Hence, antibiotic-loaded bone cement was suggested as prophylaxis for VP, especially for high-risk patients with genitourinary tract infection or patients that are immunocompromised [7,11]. For example, Opalko et al. reported that vertebral infection did not occur in a group of 50 patients, when gentamicin-loaded PMMA bone cement was used for the treatment of vertebral fractures, during the hospital stay or at the 1-year follow-up [12]. Therefore, PMMA bone cement preloaded with antibacterial agents for VP could be of substantial use to these patients. Moreover, a delayed complication of VP, adjacent-level vertebral fractures, is a major postoperative complication of this procedure [13]. It is reported that around 20 % of the patients treated with VP suffer new fractures within one year after treatment, and among them, 50–67 % occur adjacent to the treated vertebra [5]. One possible cause could be the large difference in elastic modulus between the acrylic cement and cancellous bone [14].

Previous studies have shown that the addition of small amounts of an unsaturated fatty acid, linoleic acid (LA), has great potential to improve some properties of bone cement used for VP. Typically, it has been found that after loading with max. 12 vol% LA in the liquid phase, the elastic modulus of one bone cement decreased to lower than 1000 MPa, which is a better match with the elastic modulus of human cancellous bone [15,16]. Also, the addition of LA requires a smaller volume of addition compared with other fatty acids to reach the same effect [17,18]. *In vitro* and *in vivo* studies on bone cement with the addition of LA have been conducted by using human osteoblast-like Saos-2 cells and an ovine model, showing adequate biocompatibility [[18], [19]]. A human *ex vivo* study showed that LA-loaded cement was more effective in restoring the initial mechanical properties of a fractured vertebral body compared to original cement [14].

Moreover, LA has shown good antibacterial and antibiofilm properties on its own [20]. Especially Gram-positive bacteria are susceptible to LA and the biofilm formation of *Staphylococcus aureus* (*S. aureus*) can be reduced by LA [20], probably by inhibiting the enoyl-[acyl-carrier-protein] reductase (FabI), which is an important step in bacterial fatty acid biosynthesis [21]. Furthermore, LA can also increase the membrane permeability of *S. aureus* and make it more susceptible to gentamicin [22]. Gram-negative bacteria, however, due to their different cell wall structure, are more resistant to LA [23]. It could therefore be hypothesized that the addition of LA to bone cement could not only improve the mechanical properties, but also give an antibacterial effect. However, LA is an active component in the bone cement system, and can react with the activator and initiator, resulting in the reduction of reactants available for the polymerization of methyl methacrylate (MMA) [24]. Therefore, the addition of LA to bone cement increases the residual methyl MMA monomer release, which itself has been found to reduce the viability of eukaryotic cells and bacteria [19,25,26].

Taken together, the main aim of this study was to investigate the antibacterial and antibiofilm properties of LA-loaded bone cement and to determine whether the released MMA monomer contributed to the antibacterial properties. A second aim of this study was to investigate LA release profile as well as to investigate the interaction between LA and antibiotics and the feasibility of using LA together with the antibiotic gentamicin, which is the most widely used antibiotic in bone cements, for possible synergistic effects [27].

## 2 Materials and methods

### 2.1 Material preparation

**Materials:** All chemicals were purchased from Sigma-Aldrich Co. LLC unless otherwise specified.

A commercial PMMA bone cement designed for vertebral consolidation, V-steady™ (G21 Srl, San Possidonio, Italy), hereby referred to as VS, was used as base material. The cement powder component (26.0 g) includes 54.2 % PMMA, 0.8 % benzoyl peroxide, and 45 % zirconium dioxide, and the liquid component (10.0 mL) consists of 92.6 % MMA monomer, 1.3 % N,N-dimethyl-p-toluidine, and 50 ppm hydroquinone.

A commercial gentamicin-loaded PMMA bone cement, G1A™ (G21 Srl, San Possidonio, Italy) designed for the revision of total joint arthroplasty, hereby referred to as G1A, was used as a reference. The G1A cement powder component includes 86.65 % PMMA, 1.16 % benzoyl peroxide, 9.76 % barium sulfate, and 2.44 % gentamicin sulfate, while the liquid component contains 97.5 % MMA monomer, 2.5 % N,N-dimethyl-p-toluidine, and 50 ppm methyl ether of hydroquinone.

**Bone cement specimen preparation:** The unmodified cements, VS and G1A, were prepared according to the instructions from the manufacturer, i.e., mixing the powder and liquid components manually in a glass beaker with a spatula for 30–45 s. The modified cement, VS + LA, was prepared by adding the additive before mixing the powder and the liquid. The liquid component and a vial of 1.4 mL sterile 9-*cis*,12-*cis*-linoleic acid (Evonik Industries AG, Germany) were shaken and mixed for a few seconds. After mixing, the modified cement liquid and the powder were mixed manually according to the procedure described for the unmodified cement. The resulting paste of both modified and unmodified cement was then injected into customized molds for further evaluation.

**Bacterial strains and growth conditions:** We used three strains for the characterization of biofilm formation and bacterial growth, two Gram-positive isolates of *S. aureus* and one Gram-negative *Escherichia coli* (*E. coli*), which are the main causative Gram-positive and Gram-negative bacteria in orthopedic infections [28]. A clinical gentamicin-susceptible *S. aureus* isolate (DA70300) and a clinical gentamicin-resistant *S. aureus* isolate (DA70318) from Hospital Universitario Ramón y Cajal, Madrid, Spain, were used. A uropathogenic *E. coli* CFT073 strain (DA47112), previously isolated from blood and urine, was also used [29].

Bacterial cultures were grown in Lysogeny Broth (LB). Bacterial inoculums for biofilm formation were prepared in a 96-well plate by diluting overnight cultures grown at 37 °C shaking at 190 rpm and 10,000-fold dilution with LB (approximately 10^5^ cells per well) for the incubation of biofilm on bone cement samples. Biological replicates of biofilms started from independent liquid cultures that were inoculated from independent colonies. In all microbiological experiments, three biological replicates were used.

### 2.2 Biofilm evaluation on bone cement

Bone cement was fabricated into peg shapes (approximately 17.8 mm high, top diameter 4.4 mm, surface area 45 mm^2^) that fit within commercial 96-well plates, by injecting the cement paste into corresponding molds, to follow the method published by Zaborskyte et al., using FlexiPeg biofilm device for biofilm evaluation [30].

**Biofilm growth analysis by cell viability counting:** For a direct quantification of biofilm growth on the bone cement pegs, colony forming units (CFU) from each peg were counted. At the end of the incubation (24h and 48h with a medium change after 24 h), the pegs were washed 3 × 1 min by transferring the lid with pegs to a 96-well plate containing 250 μL of sterile phosphate-buffered saline (PBS) [30]. Then the peg lid was put on the tube rack containing sterile glass tubes (15–16 mm diameter) and the pegs were pushed down into the tubes containing 600 μL PBS. Tubes with pegs in PBS were vortexed for 2 min at full speed to disperse the biofilms. CFU counts were performed by preparing serial dilutions of the bacterial dispersion in PBS and plating appropriate dilutions on LB agar plates. The experiments were done with three biological replicates and each of them had two technical replicates for plating and counting CFU per peg. For this method, if CFU per peg was less than 10^2^, which is the detection level, the CFU value would not be determined. When the CFU was above the detection level, the average CFU per peg can be calculated. (Additionally, gentamicin-susceptible and gentamicin-resistant *S. aureus* have been incubated with bone cement loading with different 3–12 vol% LA for 24h, the CFU per peg and CFU per well results are presented in the Supplementary Fig. S1. The CFU per well was done by directly taking media under the peg for serial dilution.)

**Scanning electron microscopy:** At the end of the 48h incubation, pegs were washed 3 × 1 min in 250 μL sterile PBS, and biofilms on pegs were fixed in 200 μl of 2.5 % glutaraldehyde and 1 % paraformaldehyde in phosphate buffer for 1.5 h at room temperature. Then biofilms on pegs were washed 3 × 10 min in 200 μl of phosphate buffer and dehydrated by transferring the pegs into increasing concentrations of ethanol (30 %, 50 %, 70 %, 80 %, 90 %, and 95 %) with 10-min incubation at each concentration. The final incubation in 99.5 % ethanol was continued for 20 min. The samples were air-dried and kept in a box with desiccator beads until performing scanning electron microscopy (SEM) imaging (maximum 1 day). Control pegs without any biofilm growth were also incubated for 48h in LB medium and air-dried until performing SEM imaging.

The AuPd coating of all the pegs was performed with the use of a Polaron SC7640 High Resolution Sputter Coater with 20 mA plasma current at 2 kV applied voltage for 40 s. SEM images were acquired with a Leo 1530 Gemini SEM using an acceleration voltage of 2 or 3 kV and a working distance of approximately 3 mm. One side of the peg was coated with AuPd and examined by SEM from the bottom to the top. Representative images were taken where most of the biofilm growth or typical surface morphology occurs.

### 2.3 Release of methyl methacrylate

Extracts were prepared according to ASTM F451-21 [31], for monomer release analysis of cured bone cement. Bone cement discs (diameter: 12.95 ± 0.1 mm, height: 2.00 mm ± 0.1 mm) were fabricated by injecting the cement paste into metal disc molds. After 30 min ±1 min from the onset of mixing at 37 °C, the specimens were immersed in pre-heated 5 mL deionized water, and then transferred to 37 °C for 1h, 1 day, 3 days, or 7 days. Five specimens were prepared for each time point. The diameter and height of all the specimens were recorded to calculate the surface area. After the required immersion time, 4 mL supernatant was collected. Then, 0.2 mL supernatant was diluted 10-fold with 0.04 mL internal standard solution (butyl acetate, 1000 mg/L) and 1.8 mL water. The prepared solutions were introduced into a headspace vial (V = 10 mL) and closed hermetically. The vials were incubated at 80 °C for 30 min. A trace gas chromatograph with Triplus headspace autosampler coupled to a DSQII mass spectrometer (Thermo Fisher Scientific, Massachusetts, USA) was used to measure the released MMA monomer, and 0.1 ml of the vapor phase was injected through a special syringe held at 85 °C. A ZB-624Plus column (60 m × 0.32 mm × 1.8 μm) with a helium flow of 1.8 mL/min was used for separation. The temperature program consisted of a 2 min hold at 60 °C, followed by an 8 °C/min ramp to 220 °C and a 5 °C/min hold at 220 °C. The injector, interface, and ionization source were each set to a temperature of 220 °C, 260 °C, and 200 °C, respectively. In the end, each time point had five replicate data, and the mean value and standard deviation were calculated.

### 2.4 Determination of methyl methacrylate minimal inhibitory concentration

Serial dilutions of MMA (2.5, 5, 10, 20, 40, 80, and 160 mg/mL) were done by first dissolving MMA into ethanol and then further diluting it with LB in Eppendorf tubes. This was followed by the addition of gentamicin-susceptible *S. aureus* (DA70300) cells into each tube resulting in a final volume of 1 mL containing approximately 1 × 10^5^ cells. As controls, medium containing bacteria without MMA and medium with MMA and without bacteria were used. Experiments were performed in triplicate. After overnight incubation at 37 °C with 190 rpm shaking, 100 μL suspensions from the Eppendorf tubes were transferred into a 96-well plate. OD600 was measured by a microplate spectrophotometer (Thermo Fisher Scientific, Massachusetts, USA) to indicate bacterial growth. The minimal inhibitory concentration (MIC) is defined as the lowest drug concentration, which reduces bacterial growth by more than 80 %. This experiment was performed in triplicate. The growth rate was calculated according to the following equation: Growth%= (OD_MMA well_ -OD_bacteria-free well_)/(OD_MMA-free well_ – OD_bacteria-free well_) × 100 %.

### 2.5 Release of linoleic acid

The analysis of the released LA was adapted from the monomer release analysis according to ASTM F451-21 [31]. Bone cement was prepared into disc shapes (diameter: 12.95 ± 0.1 mm, height: 2.00 mm ± 0.1 mm) by injecting the cement paste into metal disc molds. The size and weight of all the specimens were recorded. After 30 min ±1 min from the onset of mixing at 37 °C, the specimens were immersed in 5 mL pre-heated PBS and then transferred into a 37 °C oven for the following durations, 1h, 8h,1 day, 3 days, and 7 days (An alternative test was presented in the Supplementary Fig. S3, with the only difference being that extractions were collected from 1 day to 14 days with daily refreshing PBS.). There were five specimens for each time point. After the required immersion time, 4 mL supernatant was collected from each sample. The Free Fatty Acid Assay Kit (ab65341, Abcam, Cambridge, United Kingdom) was used to quantify the concentration of LA in the supernatant. The kit converts fatty acids into its CoA-derivatives which are oxidized with the concomitant generation of color or fluorescence. Following the instruction from the kit producer, 50 μL from each supernatant was directly added to a 96-well plate and palmitic acid standards with different concentrations from the kit were also prepared and transferred to the 96 well plate. First, the acyl-CoA synthesis was performed by adding 2 μL ACS reagent into each sample followed by an incubation at 37 °C for 30 min. Then, the Reaction Mix of 50 μL (45.6 μL assay buffer, 0.4 μL fatty acid probe, 2 μL enzyme mix, 2 μL enhancer) was prepared and added to each well. After incubation at 37 °C for 30 min in the dark, the fluorescent intensity was immediately measured with Ex/Em = 535/587 in a microplate reader (Infinite M200, Tecan, Switzerland). A standard curve was drawn based on the concentration of the standard solution and its fluorescent intensity. Then, the LA concentration (mg/L) of each supernatant was calculated based on the standard curve.

### 2.6 Checkerboard assay with linoleic acid and gentamicin

In order to identify the interactions of LA and gentamicin against the three bacterial strains, a checkerboard assay was conducted [32]. Gentamicin and LA stock solutions were prepared by first dissolving them into water and dimethyl sulfoxide, respectively, and then adding LB to reach the selected concentration. The two stock solutions were added into 96-well plates with multiple serial dilutions and eventually bacterial suspensions were added into each well. The final volume of each well was 100 μL with approximately 10^5^ CFU per mL. After overnight incubation at 37 °C with 190 rpm shaking, the OD600 of each well was measured, and bacterial growth was recorded. These experiments with each bacterial strain were repeated three times and each time has three replicates. The presented results were chosen from one of the representations from three repetitive experiments. The growth rate was calculated according to the following equation: Growth% = (OD_drug combination well_ – OD_bacteria-free well_)/(OD_drug-free well_ -OD_bacteria-free well_) × 100 %. The final growth % in each well was calculated by taking the average of the results from three biological replicates. Also, the MIC value of LA and gentamicin can be defined by this method. Through evaluating the combination of two compounds in increasing concentrations, this assay can provide a classification of the effect of combined compounds based on a fractional inhibitory concentration (FIC) index as follows: synergy (FIC≤0.5); additive (4>FIC >0.5); antagonism (4≤FIC) [33,34].

### 2.7 Statistical analysis

All statistical analyses and graphs were done by using the softwares Microsoft Excel and GraphPad Prism. Experimental data were presented as a mean ± standard deviation of replicate data for each sample at the tested time points or category. A significance test was performed using two-way ANOVA for the viable cell count experiment. Tukey's multiple comparisons test was used for post-hoc pairwise group comparisons with a significance level of α = 0.05. The use of two-way ANOVA was justified by the factorial experimental design, which included two independent variables (cement type and incubation time) with multiple samples per condition (n = 6).

## 3 Results

### 3.1 Bacterial biofilm formation on different bone cements

**Fig. 1:**
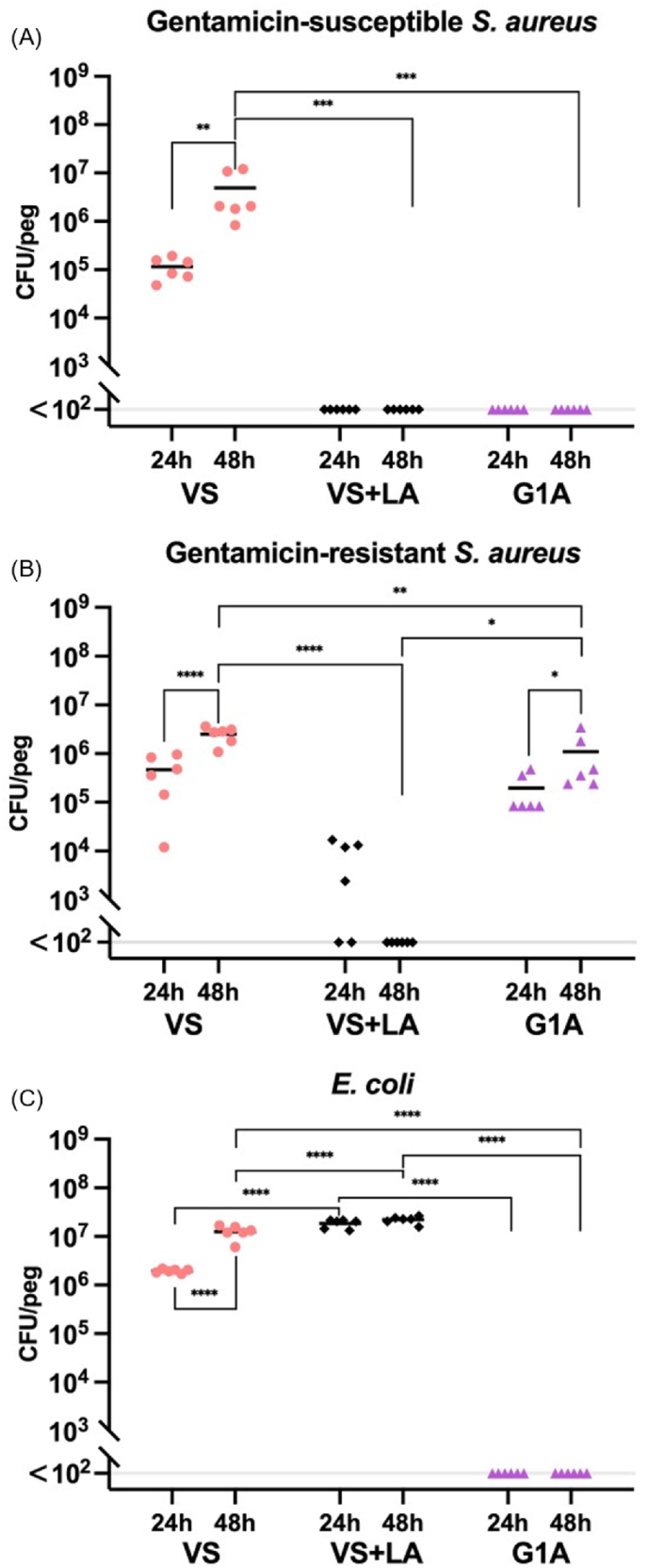
Viable cell count of three bacterial strains after 24h and 48h of biofilm growth on the VS, VS + LA, and G1A bone cement pegs. (A) Gentamicin-susceptible *S. aureus* (strain DA70300). (B) Gentamicin-resistant *S. aureus* (strain DA70318). (C) Gentamicin-susceptible *E. coli* (strain DA47112). The black line indicates the mean and the long grey line (<10^2^ CFU per peg) indicates the detection limit, i.e. no colonies were observed on agar plates after plating the lowest dilution. Statistical significance was evaluated by using a two-way ANOVA to assess the effects of different cements, incubation time, and their interaction. A Tukey's post-hoc test was used for multiple comparisons. All the comparisons having significant differences are marked on the figure. (∗ means P ≤ 0.05, ∗∗ means P ≤ 0.01, ∗∗∗ means P ≤ 0.001, and ∗∗∗∗ means P ≤ 0.0001.) A complete summary of statistical tests and results is provided in Supplementary Table S2.

**Fig. 2:**
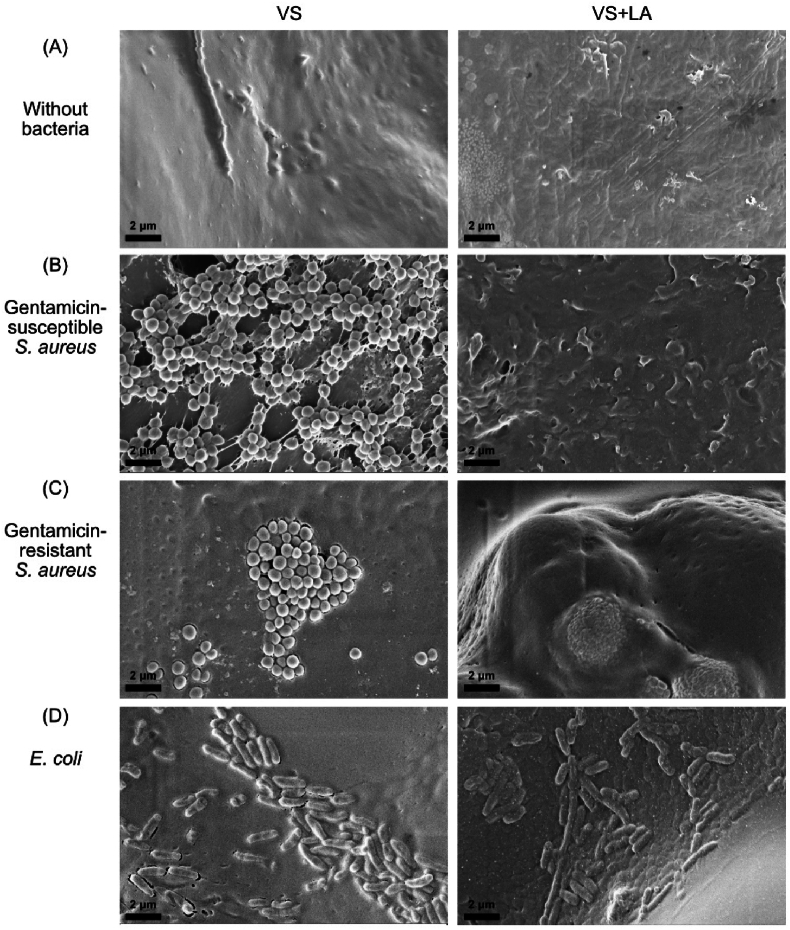
Scanning electron microscopy images of biofilms on VS (left) and VS + LA (right) bone cement pegs (A) in absence of bacteria, (B) incubated with Gentamicin-susceptible *S. aureus* (strain DA70300), (C) incubated with Gentamicin-resistant *S. aureus* (strain DA70318), and (D) incubated with Gentamicin-susceptible *E. coli* (strain DA47112) for 48h in LB medium. Images were taken at × 15,000 magnification. Scale bars are indicated on the images.

To quantify and illustrate biofilm formation on bone cements with and without addition of gentamicin and LA for the different bacterial strains, we measured CFU per peg (Fig. 1, Supplementary Tables S1 and S2) and performed SEM on the pegs (Fig. 2). Measurements of the CFU per peg on the VS cement demonstrated that all three strains formed biofilms, with 10^5^-10^6^ and 10^6^–10^7^ CFUs per peg at 24h and 48h, respectively, in the absence of any added compounds to the bone cement (Fig. 1). The results for the VS + LA cement pegs showed that LA had a significant antibiofilm effect against *S. aureus* biofilms of both gentamicin-susceptible and gentamicin-resistant strains, reducing the CFU below the detection limit (<10^2^ CFU per peg) for the *S. aureus* gentamicin-susceptible strain and to 10^3^ (24h) and under detection limit (48h) for the gentamicin-resistant *S. aureus*. Also, it was observed that the released LA from VS + LA peg suppressed the planktonic *S. aureus* in the well. However, VS + LA cement could not inhibit the growth of *E. coli* biofilms. Thus, comparisons of the antibiofilm properties of VS + LA and G1A show that G1A could inhibit the growth of gentamicin-susceptible *S. aureus* and *E. coli*, but not the gentamicin-resistant *S. aureus* for which VS + LA cement was still inhibitory.

The SEM images in Fig. 2 show biofilm growth with the gentamicin-susceptible *S. aureus* forming biofilm on the VS and VS + LA bone cement pegs. Cement pegs without bacteria are shown in Fig. 2A as a reference, and as can be noted the surface of the bone cement was relatively rough. With *S. aureus* present (Fig. 2B and C), biofilm patches and single cells formed, and the cell morphology and extracellular matrix could be seen on the surface of VS bone cement (Supplementary Fig. S2). On the surface of VS + LA bone cement, no/very limited biofilm or single cells could be found after top-to-bottom scanning of the pegs. However, it is possible that single cells could be captured in the structure due to the complexity of the bone cement surface, thereby preventing their detection. With *E. coli* present (Fig. 2D), obvious biofilm patches were found on both types of cement, showing the consistent results as CFU per peg.

### 3.2 The influence of released methyl methacrylate (MMA) on bacterial growth

**Fig. 3:**
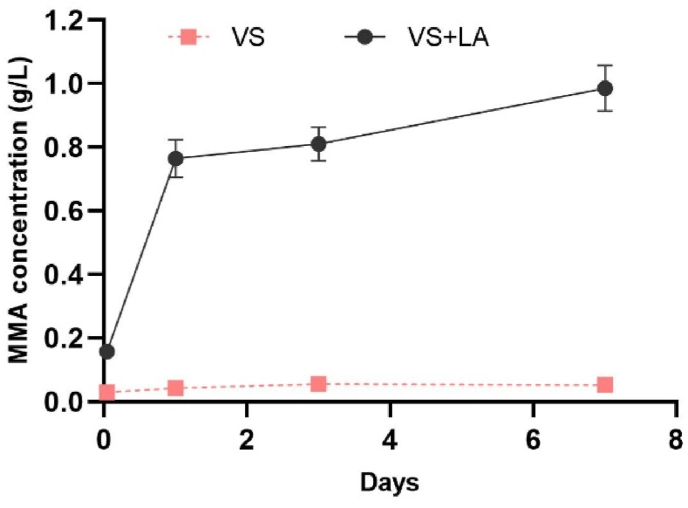
Concentration of released MMA monomer from VS and VS + LA cement samples 1h, 1 day, 3 days, and 7 days after preparation. The dot indicates the mean, and the bar indicates the standard deviation.

To investigate the influence of released MMA monomer on bacterial growth, the MIC of MMA and the released MMA concentration were determined and analyzed. The MIC of the MMA monomer against *S. aureus* (DA70300) was very high, 40 g/L, and when the MMA concentration was lower than this value there was barely no influence on bacterial growth. We compared this determined MIC value with the released MMA concentration from VS and VS + LA (Fig. 3) to assess whether the MMA could have an inhibitory effect on growth. The VS + LA bone cement released more MMA than the VS cement and the release rate was relatively fast during the first 24h, as expected [15,25]. However, after 7 days of accumulation, the released MMA from VS + LA cement reached only around 1 g/L which was still much lower than the MIC of MMA, suggesting that the released monomer had a negligible impact on bacteria growth inhibition.

### 3.3 Released linoleic acid analysis

**Fig. 4:**
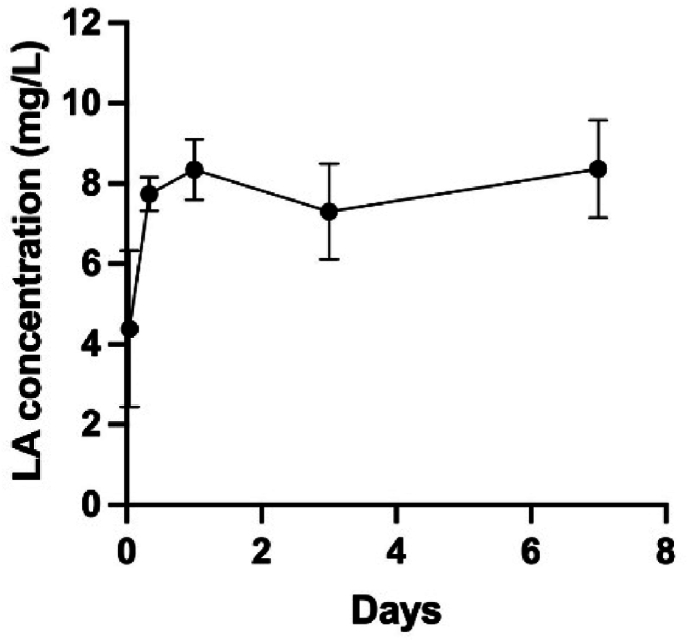
Concentration of released LA from VS + LA cement samples 1h, 8h, 1 day, 3 days, and 7 days after preparation. The dot indicates the mean, and the bar indicates the standard deviation.

To assess the LA release rate from VS + LA bone cement, the concentration of released LA was determined by using a free fatty acid assay kit. It was found that during the first 24h, LA showed a fast release and reached a concentration of around 8 mg/L (Fig. 4). After 24h, the LA concentration did not increase and remained at the same level until day 7.

### 3.4 Antibacterial interactions between linoleic acid and gentamicin

**Fig. 5:**
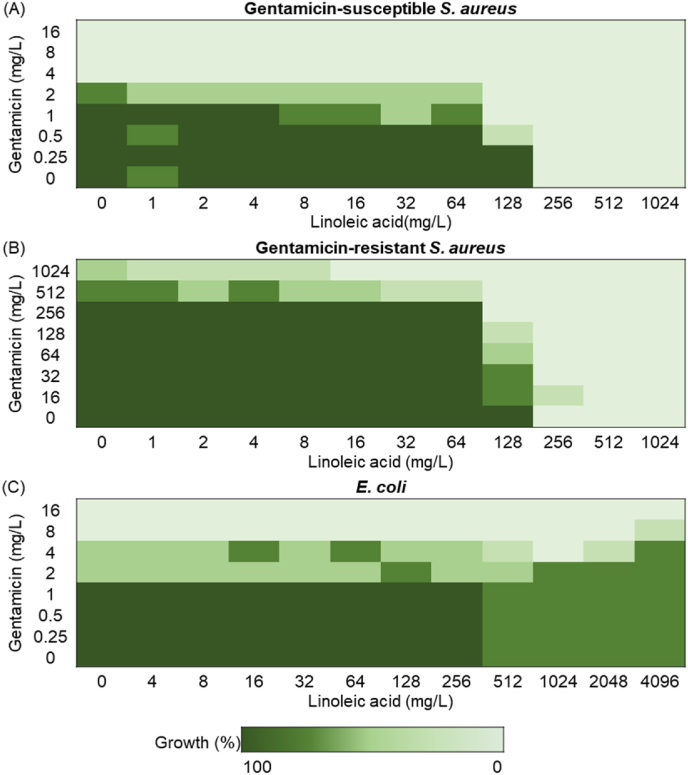
Checkerboard assay results of a combination between linoleic acid and gentamicin against (A) Gentamicin-susceptible *S. aureus* (strain DA70300). (B) Gentamicin-resistant *S. aureus* (strain DA70318). (C) *E. coli* (strain DA47112). From the lightest green to the darkest green indicates 0–20 %, 20 %–40 %, 40 %–60 %, 60 %–80 % and 80 %–100 % bacterial growth rate. (For interpretation of the references to color in this figure legend, the reader is referred to the Web version of this article.)

In order to evaluate combinations of LA and gentamicin against the three selected *S. aureus* strains, a checkerboard assay was used. This standard assay can determine the effect on bacterial growth of an individual component and the effect produced by their combination, including synergistic, additive, and antagonistic effects [32,33]. The interactions of LA and gentamicin towards the three selected strains are presented in Fig. 5. In Fig. 5A it can be seen that the combination of LA and gentamicin showed an additive effect against gentamicin-susceptible *S. aureus* (4>FIC>0.5). These results also showed that the MIC values of gentamicin and LA against gentamicin-susceptible *S. aureus* were 4 mg/L and 256 mg/L, respectively. For the gentamicin-resistant *S. aureus*, the MIC value of gentamicin was >1024 mg/L and for LA 256 mg/L (Fig. 5B). Importantly, we found that with the addition of LA, a lower gentamicin concentration was needed to reduce the growth of both strains (Fig. 5AB). For instance, using 128 mg/L LA with 1 or 256 mg/L gentamicin, respectively, inhibits the growth of gentamicin-susceptible or gentamicin-resistant *S. aureus* under the threshold of 80 % growth. *E. coli* was, as expected, not susceptible to LA [23]. Even at the LA concentration of 4096 mg/L E*. coli* still showed extensive growth (Fig. 5C) while the MIC value of gentamicin against *E. coli* was 8 mg/L.

## 4 Discussion

PMMA bone cement is a well-known and useful biomaterial but complications such as bacterial infection and adjacent-level vertebral fractures can negatively affect the treatment outcome. Adding various compounds to conventional bone cement to modify and improve its different properties is a widely considered approach. Previous studies have shown that LA is a promising additive, especially in terms of mechanical properties and handling properties: compared with the original cement, loading with LA can result in a reduction of the elastic modulus from around 2000 MPa to 600 MPa, and a reduction in the maximum polymerization temperature from 66.9 ± 2.7 to 31.1 ± 1.1 °C. Meanwhile, the setting time remained at the same level [16]. (The mechanical and handling properties of VS + LA were summarized in Supplementary Table S3.) Also, the cytotoxicity of LA-loaded PMMA cement has been evaluated in several studies using an indirect contact assay, culturing cells with cement extracts [18,35]. It showed that a four-fold dilution of the cement extract could overcome the cytotoxicity of LA-loaded PMMA cement [18]. It should be noted, however, that other base cements were used in those studies, which have shown a higher MMA release due to the addition of LA compared to the base cement used in the present study [36]. Additionally, an *in vivo* ovine study, using a base cement giving a relatively high MMA release, found no difference between standard and LA-loaded bone cement in terms of biocompatibility [19,36]. Therefore, in this study, we focused on investigating the feasibility and capacity of LA in acting as an antibiofilm agent in bone cement.

Previous studies have shown an antibiofilm capacity of LA itself against Gram-positive bacteria, especially *S. aureus*. For instance, Yuyama et al. reported that 64 mg/L LA could inhibit around 70 % of *S. aureus* biofilm formation [20], and Lee et al. found that 0.01 % LA inhibited methicillin-resistant *S. aureus* biofilm formation by more than 85 % [37]. Furthermore, a commercial bone cement, Osteopal V™ (Heraeus Medical GmbH, Hanau, Germany) loaded with 1 wt% LA (of total cement) was used to test the antimicrobial properties by an agar diffusion test [38]. This semi-quantitative assay showed that LA-loaded bone cement samples had a slightly larger inhibitory zone than the original bone cement, showing the potential of using LA in the bone cement system [38]. However, the agar diffusion test is a less precise evaluation and the antibacterial properties of LA-loaded bone cement need to be evaluated with different methods quantitatively and qualitatively.

In the current study, we assessed the antibiofilm capacity of LA-loaded bone cement by quantitatively and qualitatively evaluating the biofilm formation on peg-shaped bone cement specimens, investigating the influence of residual MMA monomer (resulting from the addition of LA) and the released LA concentration, and quantifying the ability of LA to inhibit biofilm growth alone and in combination with gentamicin when embedded in bone cement. First, we quantified biofilm formation on the surface of bone cement by using the newly-developed FlexiPeg device (Fig. 1) and qualitatively visualized the bone cement surface and the biofilm on the bone cement (Fig. 2) [30]. G1A, a commercially available gentamicin-loaded bone cement, showed as expected strong antibiofilm properties for gentamicin-susceptible *S. aureus* and *E. coli*, but not for gentamicin-resistant *S. aureus*. Most importantly, VS + LA could inhibit *S. aureus* biofilm formation, including biofilm formation by gentamicin-resistant *S. aureus*. For the gentamicin-susceptible *S. aureus*, both VS + LA and G1A could suppress biofilm formation below the detection level.

The SEM images also demonstrated the antibiofilm capacity of LA-loaded bone cement. The morphology of VS and VS + LA bone cement surfaces (Fig. 2A) was relatively rough which promotes bacterial attachment and biofilm formation [11]. This roughness might be due to an incomplete embedding process of the polymer beads within the monomer matrix, as well as the exothermic setting reaction, resulting in voids and defects on the surface [39,40]. When bone cement was incubated with *S. aureus*, the bacteria grew well on the VS surface, even in the voids, as single cells or patches (Fig. 2 and Supplement Fig. S2). However, no *S. aureus* cells were found on the VS + LA surface, in accordance with the result of viable cell count on both types of bone cements at 48h.

Based on previous findings and current results, it is conceivable that VS + LA cement could release LA to function as an antibacterial agent. The mechanism of the LA inhibitory action is not fully understood, but it could be caused by an increased bacterial membrane permeability due to the surfactant action of LA [41]. Furthermore, if LA enters the cells, it could as previously demonstrated act to inhibit FabI, an essential enzyme in bacterial fatty acid synthesis and a promising target for antibacterial agents [21]. In Gram-negative bacteria, the outer membrane appears to prevent the uptake and action of LA rendering it less effective [23], which is also consistent with our experimental results.

However, it should be noted that the addition of LA into bone cement can influence the polymerization rate by providing competing reactions to the regular propagation of MMA to the expanding chain end, resulting in shorter polymer chains and more unreacted monomers in the final material [16,24]. Since it has previously been found that monomers of MMA above 0.5 % were bactericidal to both Gram-positive and Gram-negative bacteria [26], an increased concentration of MMA due to LA addition could potentially contribute to the antibiofilm effect. To address this question, the released monomer concentrations and the MIC of MMA monomer were compared to investigate the potential effect of released MMA on bacterial growth. The MMA concentration released from VS + LA cement showed an increasing trend from 1h to 7 days, and it accumulated to approximately 0.8 g/L after 1 day and reached nearly 1 g/L after 7 days (Fig. 5) similar to previous studies [15,25]. However, the MIC of MMA was 40 g/L, which is 40-fold higher than the released MMA concentration, indicating that the released MMA from LA-loaded bone cement is very unlikely to contribute to the growth inhibitory effect.

Meanwhile, the LA release as a function of time is shown in Fig. 4. During the first 24 h, it showed a burst release. However, thereafter the LA concentration stayed at a relatively equal level until day 7. Since LA has a very low solubility in a water-based solution, the LA in the PBS extraction might reach saturation after 24h [42]. To address this, an additional experiment where the PBS was replaced with fresh PBS every 24h, was conducted and the results (Supplementary Fig. S3) indicate that VS + LA, at least until day 14, kept releasing approximately 3 mg/L LA every day. This release profile, with an initial high release in the first few hours followed by sustained low-level release for weeks, is similar to the release profile of antibiotics in some commercial antibiotic-loaded bone cements [43,44]. Bacterial adhesion, the initial step in the formation of an infectious biofilm, happens rapidly after bacteria come in contact with the biomaterial surface [10]. Therefore, having fast LA release at the beginning helps prevent biofilm formation, which also further corroborates the results that there is no *S. aureus* found on the VS + LA peg surface (Fig. 1, Fig. 2). Also, the elution duration of different antibiotics from PMMA bone cement varies from 5 days to 360 days; for example, vancomycin and gentamicin loaded cement had durations of release of 12 and 56 days, respectively [44]. The diffusion of loaded antibacterial agents is influenced by the molecular weight, the stability of the molecules in the presence of biological fluids, and the different morphology of the cement [44,45]. Thus, any future study should examine LA release and antibacterial effect over a longer time span.

Since the concentration of LA needed to inhibit bacterial growth was relatively high, we also evaluated whether LA in combination with the established antibiotic gentamicin could increase the gentamicin efficacy. Gentamicin, the most common antibiotic added to bone cement, is water soluble, has broad-spectrum antibacterial activity, and is stable towards the heat released during cement setting [27,46]. Previous studies have shown that LA exhibits synergistic effects with erythromycin against methicillin-resistant *S. aureus* [47], whereas the activity of a combination of LA and gentamicin against Gram-positive and Gram-negative bacteria is less studied [48]. Our results showed that the combination of LA and gentamicin had no antagonistic effect on *S. aureus* and *E. coli*, and against *S. aureus,* the addition of LA increased the efficacy of gentamicin (Fig. 5). Thus, a combination of LA and gentamicin could both broaden the antibacterial spectrum and increase gentamicin efficacy.

This study showed that LA is a potential antibiofilm additive to be used in the PMMA bone cement with a strong effect on Gram-positive bacteria. However, this current study is limited by the small number of tested clinically relevant strains and antibacterial agents. Although *S. aureus* is the main causative pathogen for orthopedic infection [28], future studies should consider testing other relevant clinical strains, e.g., *Pseudomonas aeruginosa.* Also, considering the limited effect on Gram-negative bacteria of VS + LA and the issue related to antibiotic resistance, future research could consider testing LA with alternative antibacterial agents to reach a broad antibacterial spectrum application.

Finally, the effect requires clinical validation, where other factors come into play. For example, the physiological fluid environment may affect the performance of the cement over time, similarly to what has been found for the mechanical properties [49].

## 5 Conclusions

Using the FlexiPeg device to evaluate the antibiofilm properties of LA-loaded bone cement, we showed that LA could effectively inhibit both gentamicin-susceptible and gentamicin-resistant *S. aureus* biofilm formation with no contribution from released non-polymerized MMA. Checkerboard assays also demonstrated good antibacterial properties of LA and gentamicin in combination, implying that LA is suitable to use together with gentamicin to prevent *S. aureus* biofilm formation and to broaden the antibacterial spectrum. Overall, LA appears as a promising additive in bone cement, where apart from improving mechanical characteristics, it can also act as an antibacterial agent.

## CRediT authorship contribution statement

**Linglu Hong:** Writing – original draft, Visualization, Methodology, Investigation, Formal analysis, Data curation. **Karin Hjort:** Writing – review & editing, Supervision, Methodology. **Dan I. Andersson:** Writing – review & editing, Supervision, Resources, Methodology, Funding acquisition, Conceptualization. **Cecilia Persson:** Writing – review & editing, Supervision, Resources, Project administration, Methodology, Funding acquisition, Data curation, Conceptualization.

## Funding sources

This research has received fundings from EIT Health (SOFTBONE, project nr 20,519), supported by EIT, a body of the European Union to CP and from the Swedish Research Council (grant 2021-02091) to DIA.

## Declaration of competing interest

The authors declare the following financial interests/personal relationships which may be considered as potential competing interests: Cecilia Persson reports a relationship with Inossia AB that includes: board membership. Linglu Hong reports a relationship with Inossia AB that includes: employment. Linglu Hong has patent #SE-21152296 pending to Inossia AB. Cecilia Persson has patent #SE-21152296 pending to Inossia AB. Cecilia Persson is cofounder of Inossia AB, a company that aims to commercialize a low-modulus cement of similar composition to the one investigated in the present study. Linglu Hong is employed part-time by the same company. If there are other authors, they declare that they have no known competing financial interests or personal relationships that could have appeared to influence the work reported in this paper.

## Data Availability

Data will be made available on request.
