# Supplementary material for: Linoleic acid addition prevents Staphylococcus aureus biofilm formation on PMMA bone cement
Source: Biofilm. 2025 Aug 7;10:100311. doi: 10.1016/j.bioflm.2025.100311 (PMC12357060; doi:10.1016/j.bioflm.2025.100311)
Supplement: Multimedia component 1 [file mmc1.pdf]

Supplementary Information

Linoleic acid addition prevents *Staphylococcus aureus* biofilm formation on PMMA bone cement

In this document, supplementary Table S1 shows the same experimental data as the "biofilm growth analysis by cell viability counting" experiment in the main text but with a different chart type to show the specific value of the mean and standard deviation of each group.

Supplementary Table S2 shows the summary of statistical analyses for the "biofilm growth analysis by cell viability counting" experiment in the main text. Supplementary Table S3 summarizes the mechanical properties and handling properties results of VS+LA from a previous publication.

Supplementary Figure S1 indicates the viable cell count results with different LA loading ratios in cement, showing that the different LA levels indicate different antibacterial property levels and that LA could inhibit the planktonic bacteria and biofilm growth.

Supplementary Figure S2 includes additional SEM images to show Gentamicin-susceptible *S. aureus* biofilms and single cells growing on VS bone cement.

Supplementary Figure S3 includes the results of released LA concentration from VS+LA cement samples from 1 day to 14 days with the replacement of fresh PBS every 24h, which is an alternative experiment of the "released linoleic acid analysis" in the main text due to the limited solubility of linoleic acid.

Strain	Cement	Time points	
		24h	48h
Gentamicin-susceptible *S. aureus* DA70300	VS	1.2×10^5 ($\pm$ 5.6×10^4)	7.3×10^6 ($\pm$ 7.8×10^6)
	VS+LA	$<10^2$	$<10^2$
	G1A	$<10^2$	$<10^2$
Gentamicin-resistant *S. aureus* DA70318	VS	4.7×10^5 ($\pm$ 3.8×10^5)	2.5×10^6 ($\pm$ 9.3×10^5)
	VS+LA	7.4×10^3 ($\pm$ 7.5×10^3)	$<10^2$
	G1A	2.0×10^5 ($\pm$ 1.8×10^5)	1.1×10^6($\pm$ 1.3×10^6)
E. coli DA47112	VS	1.9×10^6 ($\pm$ 1.8×10^5)	1.3×10^7 ($\pm$ 3.8×10^6)
	VS+LA	1.9×10^7($\pm$ 3.8×10^6)	2.2×10^7($\pm$ 3.7×10^6)
	G1A	$<10^2$	$<10^2$

Supplementary Table S1 Viable cell count of three strains for 24h and 48h biofilms grown on VS, VS+LA, and G1A bone cement pegs. (The mean of CFU/peg and standard deviation are presented. $<10^2$, indicates below the detection level).

Strain	Tukey's multiple comparisons test	Mean Diff.	95% CI of diff	Significant?	Adjusted P-value
Gentamicin-susceptible *S. aureus* DA70300	24h				
	VS vs. VS+LA	116000	-2820401 to 3052401	ns	0.9948
	VS vs. G1A	116000	-2820401 to 3052401	ns	0.9948
	VS+LA vs. G1A	0	-2936401 to 2936401	ns	>0.9999
	48h				
	VS vs. VS+LA	4920000	1983599 to 7856401	***	0.0008
	VS vs. G1A	4920000	1983599 to 7856401	***	0.0008
	VS+LA vs. G1A	0	-2936401 to 2936401	ns	>0.9999
	VS				
	24h vs. 48h	-4804000	-7342383 to -2265617	**	0.0011
	VS+LA				
	24h vs. 48h	0	-2538383 to 2538383	ns	>0.9999
	G1A				
	24h vs. 48h	0	-2538383 to 2538383	ns	>0.9999
Gentamicin-resistant *S. aureus* DA70318	24h				
	VS vs. VS+LA	458600	-504363 to 1421563	ns	0.4775
	VS vs. G1A	270000	-692963 to 1232963	ns	0.7704
	VS+LA vs. G1A	-188600	-1151563 to 774363	ns	0.8799
	48h				
	VS vs. VS+LA	2540000	1577037 to 3502963	****	<0.0001
	VS vs. G1A	1440000	477037 to 2402963	**	0.0025
	VS+LA vs. G1A	-1100000	-2062963 to -137037	*	0.0225
	VS				
	24h vs. 48h	-2074000	-2862459 to -1285541	****	<0.0001
	VS+LA				
	24h vs. 48h	7400	-781059 to 795859	ns	0.9843
	G1A				
	24h vs. 48h	-904000	-1692459 to -115541	*	0.0274
E. coli DA47112	24h				
	VS vs. VS+LA	-16660000	-20433610 to -12886390	****	<0.0001
	VS vs. G1A	1940000	-1833610 to 5713610	ns	0.4242
	VS+LA vs. G1A	18600000	14826390 to 22373610	****	<0.0001
	48h				
	VS vs. VS+LA	-9400000	-13173610 to -5626390	****	<0.0001
	VS vs. G1A	12600000	8826390 to 16373610	****	<0.0001
	VS+LA vs. G1A	22000000	18226390 to 25773610	****	<0.0001
	VS				
	24h vs. 48h	-10660000	-14128875 to -7191125	****	<0.0001
	VS+LA				
	24h vs. 48h	-3400000	-6868875 to 68875	ns	0.0542
	G1A				
	24h vs. 48h	0	-3468875 to 3468875	ns	>0.9999

Supplementary Table S2 Summary of statistical analyses for the results of viable cell count of three bacterial strains after 24h and 48h of biofilm growth on the VS, VS+LA, and G1A bone cement pegs. Two-way ANOVA was used to assess the effects of different cements, time, and their interaction. Tukey’s post-hoc test was used for multiple comparisons.

Material properties	Parameters	Data
Mechanical properties	Compressive strength	20.5 (±7.1) MPa
	Elastic modulus	559.6 (±60.3) MPa
Handling properties	Setting time	20.8 (±1.0) min
	Doughing time	9.9 (±0.1) min
	Maximum polymerization temperatures	31.1(±1.1) ˚C

Supplementary Table S3 Summary of the mechanical properties and handling properties of VS+LA cement from a previous publication.[1] Mechanical properties were measured at 24h after cement preparation with 6 replicates. Handling properties testing had 3 replicates. Mean and standard deviations are presented in the table.

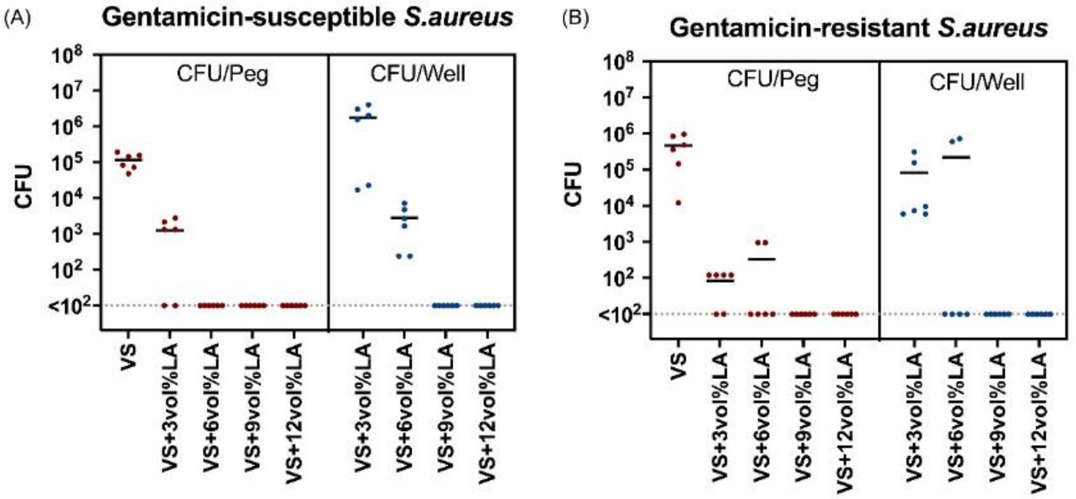

Supplementary Figure S1 Viable cell count after 24h of biofilm growth on the VS, VS+3vol%LA, VS+6vol%LA, VS+9vol%LA and VS+12vol%LA bone cement pegs and corresponding wells. (A) Gentamicin-susceptible *S. aureus* (strain DA70300). (B) Gentamicin-resistant *S. aureus* (strain DA70318).

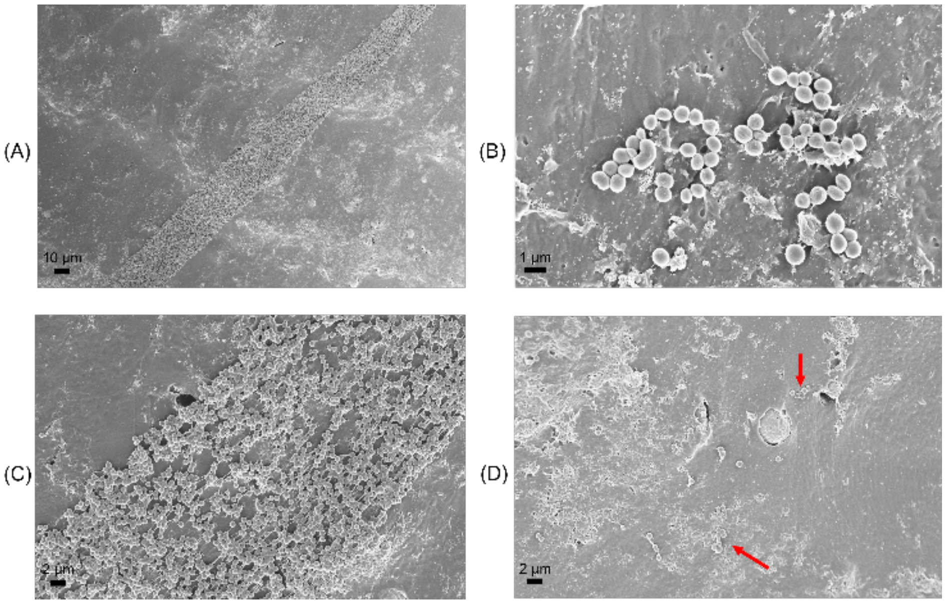

Supplementary Figure S2 Scanning electron microscopy imaging of biofilms grown for 48h on VS bone cement pegs. Gentamicin-susceptible *S. aureus* DA70300 biofilms grown in LB medium, (A) and (C) show biofilms images from the same location with different magnifications of × 1,000 and ×5,000, respectively. (B) and (D) showing single cells imaged at different locations with × 15,000 magnification and ×5,000 magnification, respectively. (Red arrows indicate the area of some single cells.)

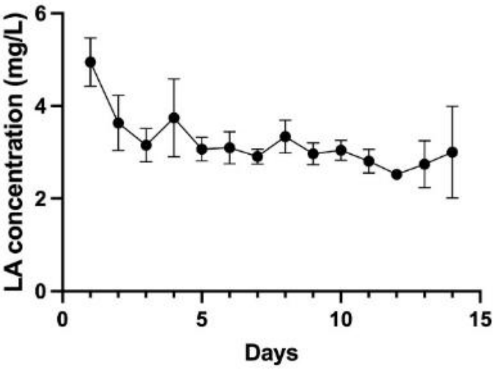

Supplementary Figure S3 Concentration of released LA from VS+LA cement samples from 1 day to 14 days after preparation, with replacement of fresh PBS every 24h to collect extraction. The dot indicates the mean, and the bar indicates the standard deviation.

Reference

(1) Robo, C.; Wenner, D.; Ubhayasekera, S. J. K. A.; Hilborn, J.; Öhman-Mägi, C.; Persson, C. Functional Properties of Low-Modulus PMMA Bone Cements Containing Linoleic Acid. *J Funct Biomater* **2021**, *12* (1), 5. https://doi.org/10.3390/jfb12010005.
